# Establishment of hospital resilience framework in urban China: insight from Wuhan City

**DOI:** 10.1007/s43762-022-00060-z

**Published:** 2022-09-15

**Authors:** Annan Jin, Gang Li, Yue Yu, Jiaobei Wang, Qifan Nie

**Affiliations:** 1 College of Urban and Environmental Sciences, Northwest University, 710127 Xi’an, China; 2 Shaanxi Key Laboratory of Earth Surface System and Environmental Carrying Capacity, 710127 Xi’an, China; 3 Alabama Transportation Institute, 248 Kirkbride Lane, Tuscaloosa, AL 35487 USA

**Keywords:** Hospital resilience, COVID-19, Response framework, Spatial analysis, Optimization

## Abstract

Since the Corona Virus Disease 2019 (COVID-19) swept the world, many countries face a problem that is a shortage of medical resources. The role of emergency medical facilities in response to the epidemic is beginning to arouse public attention, and the construction of the urban resilient emergency response framework has become the critical way to resist the epidemic. Today, China has controlled the domestically transmitted COVID-19 cases through multiple emergency medical facilities and inclusive patient admission criteria. Most of the existing literature focuses on case studies or characterizations of individual facilities. This paper constructs an evaluation system to measure urban hospital resilience from the spatial perspective and deciphered the layout patterns and regularities of emergency medical facilities in Wuhan, the city most affected by the epidemic in China. Findings indicate that the pattern of one center and two circles are a more compelling layout structure for urban emergency medical facilities in terms of accessibility and service coverage for residents. Meanwhile, the Fangcang shelter hospital has an extraordinary performance in terms of emergency response time, and it is a sustainable facility utilization approach in the post-epidemic era. This study bolsters areas of the research on the urban resilient emergency response framework. Moreover, the paper summarizes new medical facilities’ planning and location characteristics and hopes to provide policy-makers and urban planners with valuable empirical evidence.

## Introduction

The outbreak of novel coronavirus pneumonia has posed a serious threat to social and economic development and has been defined as a severe global public health emergency (Peng, 2020; Lauer et al., 2020; Zhou et al., 2020). Due to the 2019 novel coronavirus (2019-nCoV) can be transmitted through droplets and close contact, with an incubation period of 1 to 14 days (Huang et al., 2020). The virus spreads worldwide at a rapid rate, including in China, experiencing an unprecedented lack of medical resources and hospital beds (Chen et al., 2020a; Fang et al., 2020). So far, many countries have still suffered from the high epidemic period.

Today, public health events with substantial impacts are caused mainly by infectious diseases, such as SARS, Ebola, and COVID-19 (Woods et al., 2020). There is a reason why the urban medical response framework is being so severely challenged by public health events—the medical facilities not only treat normal cases, but accept a lot of confirmed cases (Xiao et al., 2020). Therefore, it is difficult for extant existing medical facilities hospitals to control the epidemic, and it is urgent to establish new types of medical facilities. From a resilient perspective, the effects of the medical response framework are closely related to response time and cooperation between different medical facilities (Tyler & Moench, 2012). Thus, the evaluation studies of the representative city for medical response framework would improve an understanding of the current resilience level of city medical facilities against pandemic disasters for city planners, policy-makers, and managers.

Wuhan, most severely affected by the epidemic in China, has also faced the same challenge. However, the epidemic has been controlled in only 4 months. Not only have control policies been implemented, but a resilient emergency medical response framework has been established in a short time. Three types of emergency medical facilities, the designated hospitals, the Fangcang shelter hospitals, and the makeshift hospitals, have been immediately set up after the outbreak (Li et al., 2021a; Zhou et al., 2022). Confirmed cases of different conditions have been assigned to different types of emergency medical facilities based on the specific admission criteria. To learn the resilience and effectiveness of the medical response framework. The paper primarily measures the emergency timeliness, spatial accessibility, and service coverage of medical facilities and from a set of crucial function characteristics and indexes, including spatial pattern, bed number, construction speed, and location planning. Besides, to be following the fact of patients’ medical demands, the evaluation indexes are considered with the specific admission criteria during the epidemic.

In the past few years, most authors have just focused on the architectural features and their control effects of Fangcang hospitals but the few studies attention to urban hospital resilience response framework (Shang et al., 2020; Juan et al., 2020; Wang et al., 2020). Moreover, the paper studies the planning and location characteristics of new types of medical facilities, and hopes to provide policy-makers and urban planners with valuable empirical evidence.

This article will be divided into the following sections. The second part first reviews the related work. The third part formulates the evaluation framework of hospital resilience and introduces the relevant indicators. The fourth part explains the data source, discusses the definition of index, and uses the spatial analysis method in this paper. The fifth section introduces the research results. Finally, this paper puts forward some policies and suggestions on the layout of emergency medical facilities in response to similar disasters in the future.

## Literature review

There is an increased risk of natural disasters and public emergence in urban. The concept of resilience gradually comes to be familiar with us, and it can be encompassed the qualities that enable the individual, organization, or community to resist, respond to and recover from the impact of disasters .(The concept of resilience, 2021; McAslan, 2010). From the urban resilience perspective, the adopted definition of resilience focuses on how a city can absorb and adapt to external pressures during any crisis, hazards or disasters (Rus et al., 2018) The hospital resilience is essential in the urban resilience because it provides ‘lifeline’ services to minimize the impact of disasters on the community and achieve higher urban resilience. It has gained increasing interest in relevant studies. Developing the concept of “hospital resilience” will provide a starting point for agreement about what it comprises and how to measure it. The definition of hospital resilience, rooting in the urban resilience, can be understood as the ability of hospitals to resist, absorb, and respond to the shock of disasters while maintaining and surging essential health services, and then to recover to its original state or adapt to a new one (Zhu et al., 2019). It has four key criteria, namely, robustness, redundancy, resourcefulness and rapidity (Zhong et al., 2014). These criteria match hospital safety, disaster preparedness, resources, continuity of essential medical services, recovery, and adaptation (Zhong et al., 2014).

The comprehensive evaluation indexes of the hospital resilience can contribute to establishing the framework and the methods used by questionnaire survey and statistical analysis (Chen & Quan, 2021; Chu et al., 2021; Jolgehnejad et al., 2020; Verheul & LA Dückers, 2020). At present, the evaluation of hospital resilience is mainly carried out from the aspects of construction engineering and hospital management (Jolgehnejad et al., 2021; Cimellaro et al., 2018). The indicators for evaluating hospital elasticity in the engineering field are mainly structural indicators (such as electricity, water supply and transportation networks) and non structural indicators (such as communication systems, gas supply systems, sewage systems, etc.) (World Health Organization, 2010; Fallah-Aliabadi et al., 2020) the report of the Pan American Health Organization (PAHO) shows that non structural components usually account for more than 80% of the total cost of hospitals. Most studies are based on different natural disasters and hypothetical scenarios (Achour et al., 2014; Radovic et al., 2012; Simonovic & Peck, 2013). By determining the elastic range of various indicators, we can ensure that the hospital can operate normally and have a certain flexibility in case of emergency. Grimaz developed the radar-hf method (reconnaissance analysis for detecting actual situations and improving requests, which is applicable to hospital facilities). Radar hospital facility (radar-hf) is a multifaceted method for hospital physical environment situational assessment. By evaluating the robustness, redundancy, resourcefulness and rapidity of the status of different hospital facilities, decision makers can plan modernization strategies and flexible improvements (Grimaz et al., 2021).

On the other hand, some studies put forward the evaluation framework of hospital resilience from the perspective of hospital management, (Chen et al., 2021; Shirali et al., 2016; Tharanga & Fernando, 2021) such as evaluating the relevant indicators in the four dimensions of technology, organization, society and economy, and considering two types of costs related to hospital resilience: (1) systematic impact of disruption which shows the differences of performance index before and after disasters, and (2) total recovery effort which indicates the costs of recovery action that would be done after disruptions to return the system state to pre-disruption one.(Renzetti & Mango, 2020; Moreno-Leal et al., 2021; Pacheco, Martimbianco, Roitberg, et al, 2021) Shuang Zhong,(Zhong et al., 2015) based on the modified Delphi, proves that equipment for on-site rescue, plan initiation, equipment for referral of patients with complex care needs are crucial indicators of a comprehensive framework of hospital resilience. Meanwhile, the individual resilience of hospital staff also is considered as an influencing factor of hospital resilience. Maria Mourinho de Almeida(Moitinho de Almeida et al., n.d.) undertakes a qualitative study with Semi-structured interviews in hospital staffs and shows that hospital resilience and individual staff resilience are interdependent.

The ongoing global spread of the COVID-19 pandemic presents challenges to hospital resilience as well as opportunities and empirical data to study hospital emergency response-ability. The scale and frequency of emerging infectious diseases are increased mainly to population mobility, social contact, convenient transportation, and other factors (Li et al., 2021b; AbouKorin & A., Haoying Han, and Mahran Gamal N. Mahran., 2021; Wang et al., 2021). Therefore, the resilience framework further incorporates empirical evidence on resilience against pandemic disasters. Jie Chen selected the population inflow from the covid-19 epidemic epicenter, urban population density and city size (Ali et al., 2021). In order to analyze the relationship between these factors and city-level resilience against the outbreak of the COVID-19 epidemic. The hospitals resilience framework has been used as a decision support tool to increase the resilience index of systems under natural disasters. It can be achieved through the “PPRR” management strategy of prevention and mitigation (P), preparation and planning (P), response and relief (R), and recovery (R) (Chatterjee & Mukherjee, 2013).

In summary, previous studies mainly focus on medicine, management, and engineering and give insights into the hospital resilience’s definition and evaluation framework. However, a minority of studies explore how the distribution and planning of hospitals correlate with an urban controlling the epidemic from a space perspective. Does this perspective reflect how the functions, distribution, and serviceability of emerging hospitals resist epidemic outbreaks from a macroscopic level? This paper hopes to bridge this gap.

## Evaluation framework and indicators

**Fig. 1:**
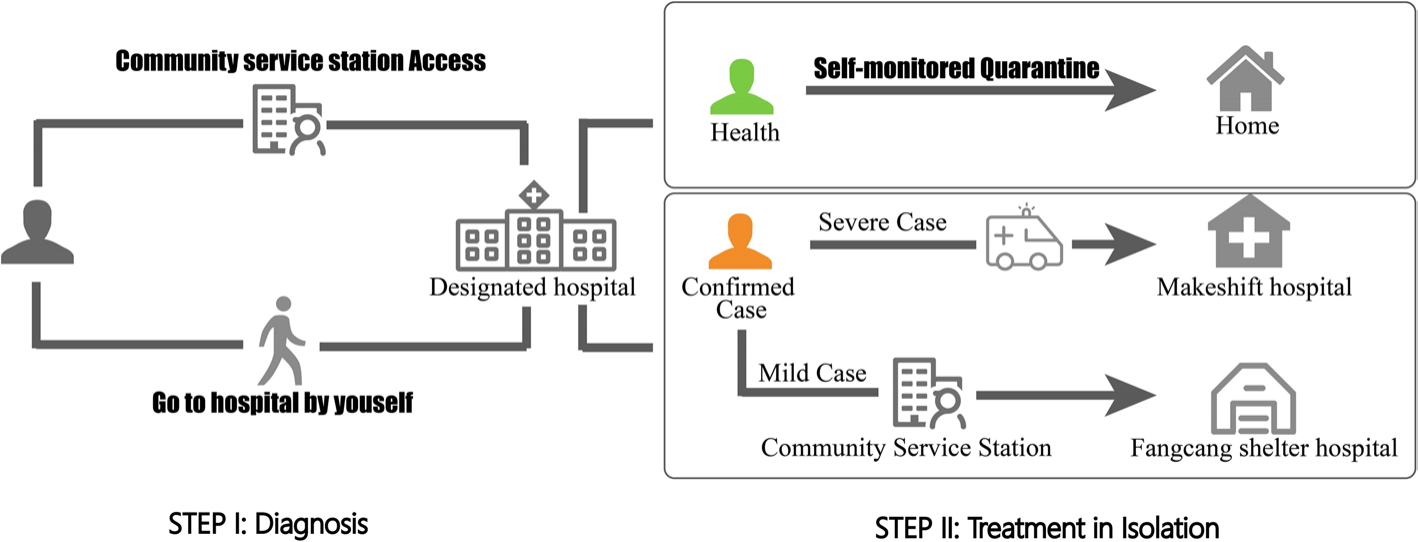
The medical treatment process during the COVID-19 epidemic in Wuhan

To establish the evaluation framework and indicators of hospital resilience from a spatial perspective, it is necessary to clarify the functions and status characteristics of emergency medical facilities, The three aspects: medical-grade, facility capacity, and accessibility are usually considered as the key indicators in general hospitals’ location evaluation from a space perspective (Bajow & Alkhalil, 2014; Eldemir & Onden, 2016; Kumar et al., 2016; Arnolds & Gartner, 2018). Also, influenced by the market economy and residents’ demand. The other factors such as competitors, environmental pollution, and land use also become evaluation indicators.(Zhang et al., 2020) However, emergency medical facilities are different from ordinary hospitals in function and status. They are responsible for treating critically ill patients and dealing with emergency disasters and public health events. Therefore, it is a significant part of establishing the hospital resilience framework. Meanwhile, the evaluation indicators have to consider specific admission criteria during the epidemic because specific prevention policies, such as lockdown, insulating treatment, and triage patients, make residents’ medical treatment demands different (World Health Organization, 2020; Ung, 2020). According to the Notice on Further Standardizing the Admission of Patients to Designated Hospitals for Pneumonia with Novel Coronavirus Infection published by the Wuhan Health Commission,(Wuhan Health Commission, 2020). The first is testing, where all suspected patients will undergo nucleic acid testing (NAT) in the designated hospital. The second is treatment. If the patient is diagnosed, the designated hospital will transfer to other medical charities according to the patient’s condition (Fig. 1). Generally, the mild patients will be sent to the Fangcang Sheltered Hospital, and the severe patients will be sent to two makeshift hospitals (Huoshenshan Hospital and Leishenshan Hospital). In this paper, the designated hospitals, approved by the government and other departments, refer to the hospitals that specialize in testing and treating citizens who are confirmed with COVID-19. The makeshift hospitals refer to temporary specialized hospitals that admit patients diagnosed with infectious diseases specified in the Law of the People’s Republic of China on the Prevention and Control of Infectious Diseases in emergencies. The Fangcang shelter hospitals refer to temporary treatment places where the government epidemic prevention and control emergency command temporarily commandeer existing social buildings, such as gymnasiums, exhibition halls, warehouses, and other tall spaces, for centralized admission of patients with minor illnesses (Group standards of the People 's Republic of China ( T / UPSC 0001–2020 ), 2020).

Therefore, in combination with the functions of emergency medical facilities and taking into account the medical rules of residents in Wuhan during covid-19, we constructed the evaluation framework of hospital resilience from the perspective of facility spatial distribution and residents’ service demand. Rapidity has been proved by many studies to be one of the important criteria for assessing hospital resilience.

### Emergency timeliness

Rapidity reflects the response speed of medical facilities to disasters, and to a certain extent reflects the disaster preparedness capacity and resources of medical facilities. Therefore, this paper first evaluates the emergency timeliness of various medical facilities in Wuhan. The main evaluation index used in this part is the occupancy rate of hospital beds (Li et al., 2021d; Hick et al., 2009). The emergency timeliness hopes to evaluate the disaster preparedness and resources in hospitals. On the one hand, hospital bed-occupancy rates have been proposed as a measure of the ability of a hospital to function safely and effectively (Borg, 2003; Kibbler et al., 1998; Borg et al., 2008; Cooke et al., 2004; Keegan, 2010; Chi et al., 2017). A study by Keegan, Andrew D shows that high bed-occupancy rates are associated with greater risks of hospital-associated infection and access block and have a negative impact on staff health. Therefore, reducing the emergency scheduling time requires more resources and costs.(Chi et al., 2017). This study uses the number of total beds and remaining beds of designated hospitals to analyze the trend of changes in medical resources. On the other hand, most emergency hospitals were changed from general hospitals. Emergency hospitals’ opening time reflects the ability to recover and adapt to urban hospitals. Therefore this study compares the medical facilities’ construction and opening time with beds number change trend changing in time series.

### Spatial accessibility

Under the influence of urban population growth, road congestion and medical facility level, the spatial accessibility of emergency medical facilities determines the convenience of residents to the facilities in case of emergency. Accessibility of emergency medical facilities is essential for service quality and satisfaction and is one of the fundamental requirements of residents (World Health Organization, 2020). It represents convenience of reaching the service facilities at the spatial scale of demand. The spatial accessibility of facilities is generally used for the location of facilities and the evaluation of service capacity. Most of the current evaluation methods consider the road distribution of the city and the attractiveness difference caused by different levels of facilities. Generally, the hierarchical two-step floating catchment area (H2SFCA) method and weighted average travel time are used for calculation (Luo & Wang, 2003; Yang et al., 2006; Yin et al., 2018; Gutiérrez, 2001). Assessing the spatial accessibility of emergency medical facilities can further optimize the rationality of the layout of facilities, and is helpful for the adjustment of the level and quantity of emergency medical facilities. This paper mainly selects the urban road density index and the facility accessibility index based on the weighted average travel time.

### Services capabilities

**Fig. 2:**
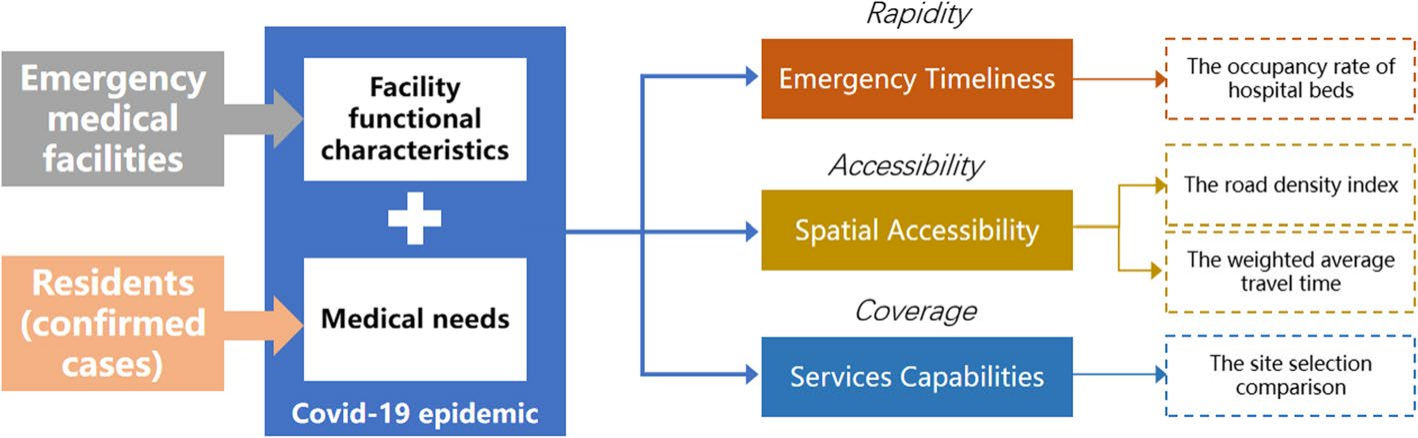
The evaluation framework and indicators

Wuhan responded to the shortage of hospital beds during the covid-19 epidemic by rebuilding gymnasiums and Exhibition Center facilities in the city as shelter hospitals. As a new type of emergency medical facilities, the shelter hospital is also rebuilt based on the existing facilities in the city. At present, few studies focus on the specific service scope and capacity of the shelter hospital. During the covid-19 epidemic, the confirmed cases in Wuhan were assigned to the designated shelter hospital according to the community where the residents lived. Therefore, the service coverage can reflect influencing factors of setting up the emergency facilities from a macro level and spatial perspective and reference when other cities face similar challenges This paper calculates the collected urban public facilities (public gymnasium, University Gymnasium, convention and Exhibition Center, conference center, etc.) by setting different impedances (time and distance) and using the maximum coverage model, and compares the results with the actual locations of 13 shelter hospitals in Wuhan, so as to estimate the service capacity and scope of emergency hospitals (Fig. 2 and Table 1).

**Table 1:** Evaluation indicator table

First-level indicators	Second-level indicators	Support data
Emergency Timeliness	The trend of the designated hospitals’ bed number	Number of total beds, Remaining beds, Opening times of emergency medical facilities
c	Road network density	Hospital grade, Urban road network data, Bed number of emergency medical facilities
	Weighted average travel time	
Facility Coverage’ Degree	Facilities’ number in different region	Administrative Region Data, Public Facilities POI Data, Facilities Service radius
	Facility service coverage	

## Data and method

### Data

**Fig. 3:**
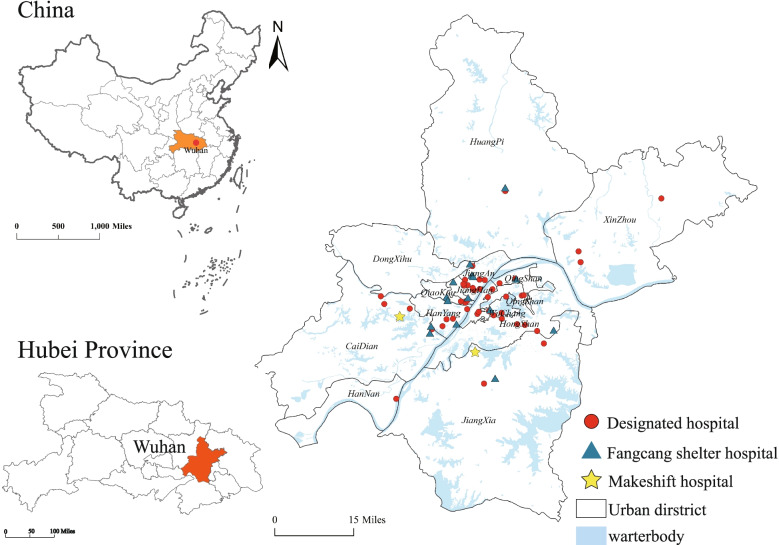
Study area and the emergency medical facilities in Wuhan

Wuhan is the capital of Hubei Province, located in the eastern Jianghan Plain, and the middle reaches of the Yangtze River, covering an area of 8569.2 km^2^ with a population of 11.2 million by the end of 2019 (Wuhan Statistics Bureau, 2020). It is the central city of the urban agglomeration in the middle reaches of the Yangtze River and one of the nine National Central Cities of China, leading the development of Central China (Fig. 3). As of January 30, 2021, there were 50,340 confirmed cases in Wuhan, the most severe city affected by COVID-19 in China. The data for this study were obtained from the list of emergency medical facilities and data on the number of daily bed changes in the designated hospitals published by the Wuhan Health Commission (1150 entries, as of February 25, 2020, http://wjw.wuhan.gov.cn/), and from Baidu Maps (https://map.baidu.com/) for 62 the location of emergency medical facilities (including 45 designated medical facilities, two makeshift hospitals, and 15 Fangcang shelter hospitals), 45 public facilities and 27,590 other related the point of interest (POI) data (including 27,473 in residential communities and 117 in community service centers). The data on area, resident population, population density, number, and grade of hospitals in each administrative region were collected from the Wuhan statistical yearbook of 2019 (Wuhan Statistics Bureau, 2020).

### Methodology

ArcGIS was used to visualize the emergency medical center’s spatial pattern and service area, and SPSS was used to evaluate the emergency timeliness and coverage. The indicators are as follows.

(i) Pearson correlation

Correlation coefficient can accurately indicate the close relationship between the two variables, in order to explore relationship between the number of facilities and influence factors using SPSS 22.0 software. Pearson’s correlation coefficient is the covariance of two variables divided by the product of their standard deviations, which displays the degree of linear correlation between variables. Pearson correlation coefficient is also known as Pearson product-moment correlation coefficient which reflects the relationship between two variables., the formula is presented below,

1 $${r}_{xy}=\frac{n\sum XY-\sum X\sum Y}{\sqrt{\left[N\sum {X}^2-{\left(\sum X\right)}^2\right]\left[N\sum {Y}^2-{\left(\sum Y\right)}^2\right]}}$$

Where *n* is the sample size; *X* and *Y* are the observed values of the variables. The correlation coefficients are less than or equal to 1, where 1 is total positive linear correlation, 0 indicates no linear correlation, and − 1 is total negative linear correlation. In this paper, Pearson correlation equals to 1 is used to determine the relationship between the number of facilities and influence factors. Where *n* is the sample size, which represents the observations and mean values of the two variables, with a range of values of 1, − 1, and a value of 1

(ii) Curvilinear regression model

The model allows the transformation of non-essential linear relationships into linear relationships through variable transformation to describe the quantitative relationships between variables. The main methods are the curve linearization method and the curve-fitting method. The curve fitting method can require the explanatory variables of certain time measures, and by solving the curve regression equation of the curvilinear relationship between variables, the curve regression equation with better fit is selected for fitting analysis. The commonly used regression model is the compound curve, quadratic curve, and cubic curve (Song & Faming, 2014). This study used a regression model to analyze the trend of the facilities’ bed number.

(iii) Weighted average travel time

The index evaluates the travel time from the attraction to the center point, thus reflecting the accessibility of the attraction. It is mainly determined by the center point’s spatial location and is also related to the size class of the attraction point. The lower the index score, the higher the accessibility of the point, and vice versa (Minghua, 2005). This is a useful indicator in evaluating the accessibility of facilities at different levels, the formula is presented below,

2 $${t}_i=\sum_j{p}_{ij}{t}_{ij}$$

Where *t*_*i*_ is the weighted average travel time of the center point; *p*_*ij*_ is the travel probability from the center point to the attraction point, and the formula is presented in (3); *t*_*ij*_ and is the actual travel time of the center point.

3 $${p}_{ij}=\frac{{}^{{m}_j}\!\left/ \!_{{d}_{ij}^{\alpha }}\right.}{\sum_j\frac{m_j}{d_{ij}^{\alpha }}}$$

Where *m*_*j*_ is the size of the attraction site (this study uses the number of beds in the emergency medical facility); *α* is the decay index, which is generally 1.0–3.0 (this study takes 1.0); *d*_*ij*_ is the distance from the center point to the attraction point.

(iv) Maximum coverage model

The maximum coverage model considers the allocation model of existing facilities with specific distance requirements, (Minghua, 2005; Hakimi, 1965; Toregas et al., 1971) it was proposed by Church (Church & ReVelle, 1974) based on the P-center model and the centralized coverage model. The objective is to select the spatial location of a set number of facilities among all candidate facility sites so that the most demand points are located within the maximum service radius of the facility site, mainly for an emergency, disaster prevention and other security facilities with mandatory service radius restrictions that are funded by the government. This study uses the location assignment tool in Arc GIS to construct this model.

## Results

This section first uses the spatial analytical methods in order to learn about the spatial pattern of EMS hospitals. Then evaluates and explains the indicators including the emergency timeliness, the spatial accessibility and the service coverage.

### Spatial pattern

**Fig. 4:**
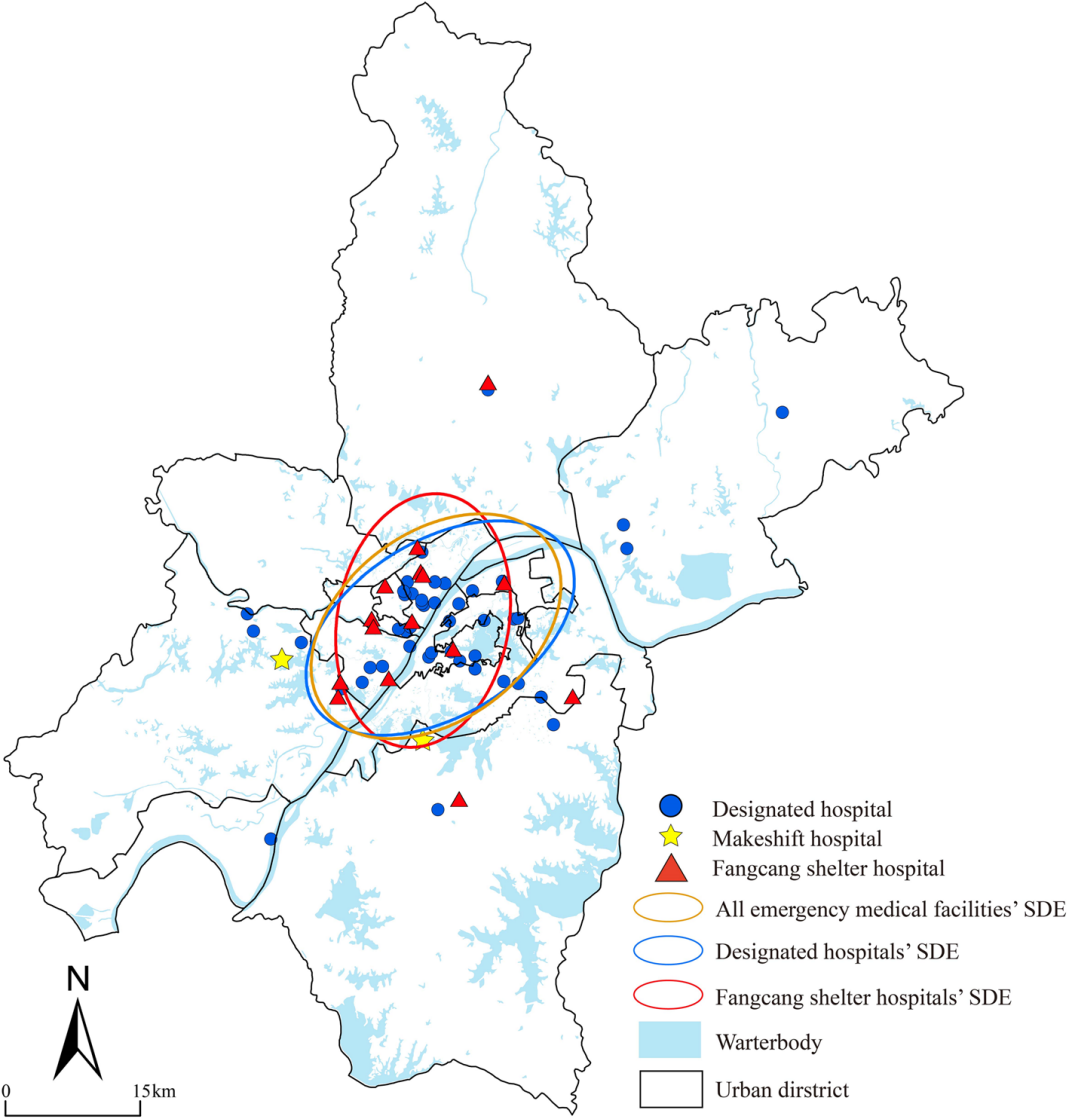
The Layout of Emergency Medical Facilities in Wuhan

The spatial pattern help to learn about the distribution directions and cluster or scatter features of EMS hospitals. The results of the standard deviation ellipse (SDE) analysis for all facilities show that the spatial layout is symmetrically distributed along the axis of “northeast-southwest.” The mainly reason is that the Yangtze River and the Han River through the city influence the urban form. Furthermore, using the average nearest neighbor index (ANNI), the cluster analysis shows that the nearest neighbor index of the emergency medical facilities of two types were (excluding only two makeshift hospitals): the designated hospitals are 0.564, and the Fangcang shelter hospitals are 1.394. This means that the designated hospitals show a clustered distribution, while the Fangcang shelter hospitals are the opposite. Thus, all facilities formed a pattern that was clustering in center and scattering in margin (Fig. 4). These cluster areas result both the designated hospitals are mainly located in the main urban area and their number accounts for 72.58% of the total number of EMS hospitals. Besides, the 2019-nCoV could transmit by respiratory droplet and contact. The EMS hospitals which are located in the margin could satisfy demands of suburb residents and prevent the virus second spreading.

### Emergency timeliness evaluation

**Fig. 5:**
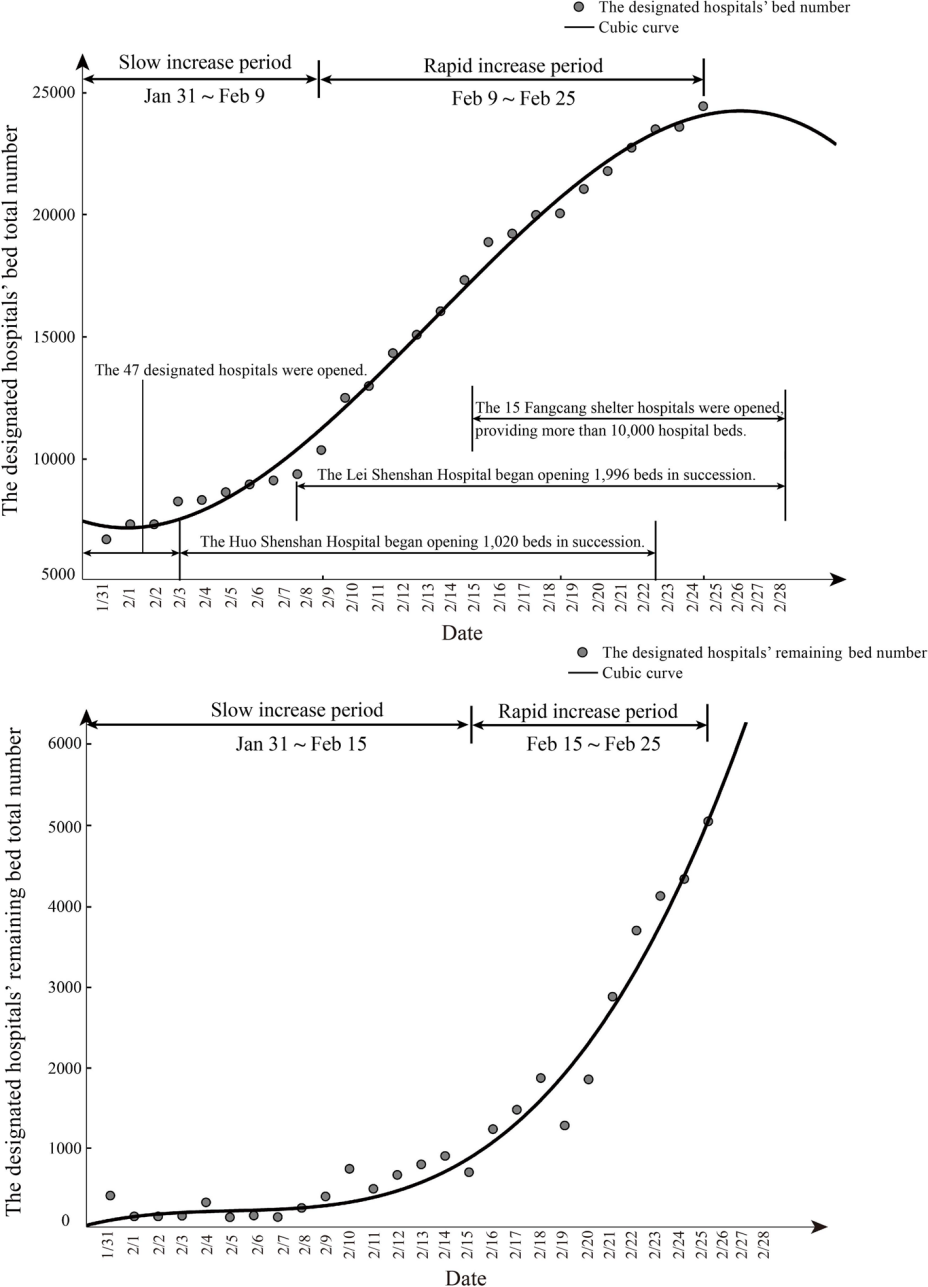
The relationship between the number of beds and the opening hours of hospitals

Hospital bed-occupancy rates have been proposed as a measure of the ability of a hospital to function safely and effectively (Chen et al., 2020b). It is limited by the type of data published by Wuhan Health Commission. In this study, the total and remaining number of beds in designated hospitals during Jan 31, 2020 and Feb 25, 2020 were used as research data and were compared their changing trends to reflect the ability in recovery and adaptation of urban hospitals. The curvilinear estimation results of bed number trends show that the *p*-values of all three regression models are less than 0.01, also the third curve model was the best fitting results with fitted values R^2^ of 0.994 and 0.972. Therefore, we use the third curve model to analyze the trend of the bed number change (Fig. 5).

The result showed that the two types of data both had the same changing trends that were rapid increase period and slow increase period. However, there were differences in aspects of speed and start date. In the Slow increase period. The increase in the total number of hospital beds lasted from Jan 31 to Feb 9, with an average of 365.9 new beds per day to 9312 beds. Moreover, the number of remaining beds was also insufficient at the beginning and showed slow increase characteristics from Jan 31 to Feb 15. In this period, the number of confirmed cases rapidly increased, while the new makeshift hospitals were just gradually operational, and the renovation of the Fangcang shelter hospitals just started. This phenomenon was alleviated when the Fangcang shelter hospitals were put into operation after Feb 15. In the rapid increase period, the total number of hospital beds and the number of remaining beds showed a rapid increase from Feb 9 to Feb 25. Furthermore, the number of remaining beds increased more rapidly than total number of hospital beds, mainly because the two makeshift hospitals were fully operational after Feb. 9, and started treating severe patients. In addition, the shortage of beds was eased by the use of 15 Fangcang shelter hospitals after Feb 15.

### Spatial accessibility evaluation

#### Traffic environmental evaluation

**Fig. 6:**
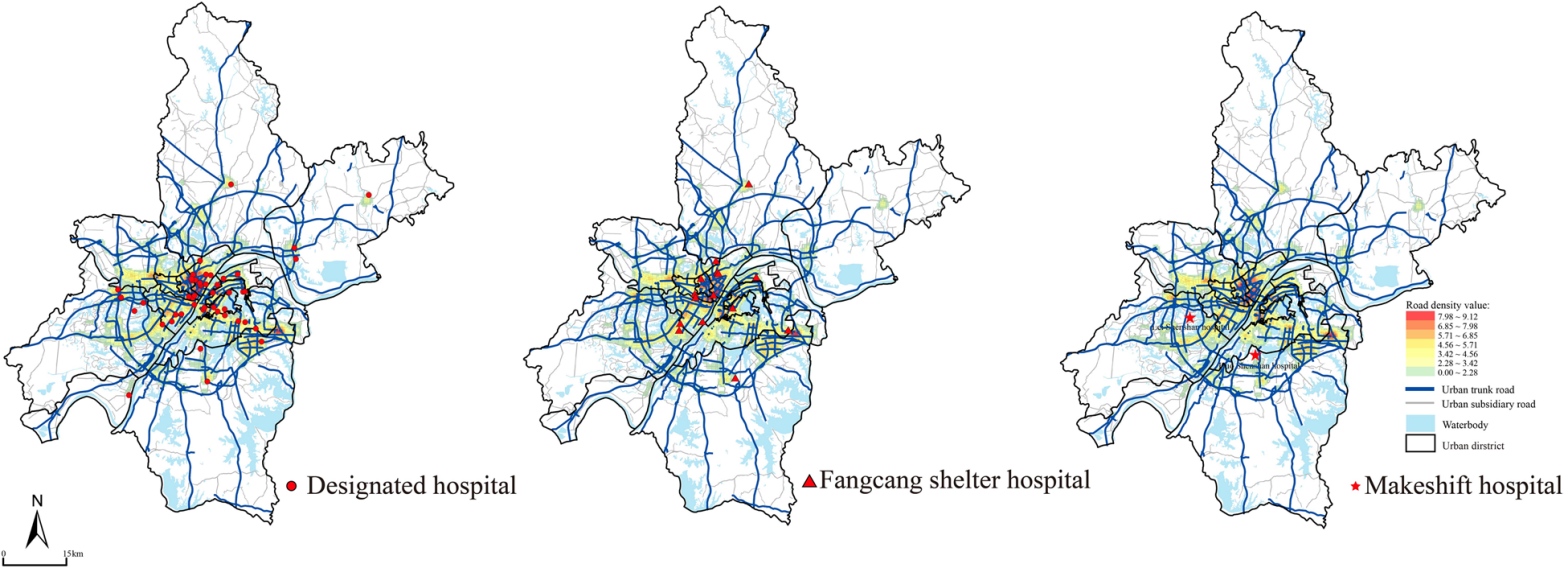
The relationship between three emergency medical facilities and road environment

The road network density map shows that both the designated hospitals and the Fangcang shelter hospitals are laid out along the main roads in high-density areas. Although the makeshift hospitals are laid out in the suburban districts, they are surrounded by highways and urban expressways, facilitating the transfer of severe patients and medical supplies (Fig. 6).

#### The result of weighted average travel time

**Fig. 7:**
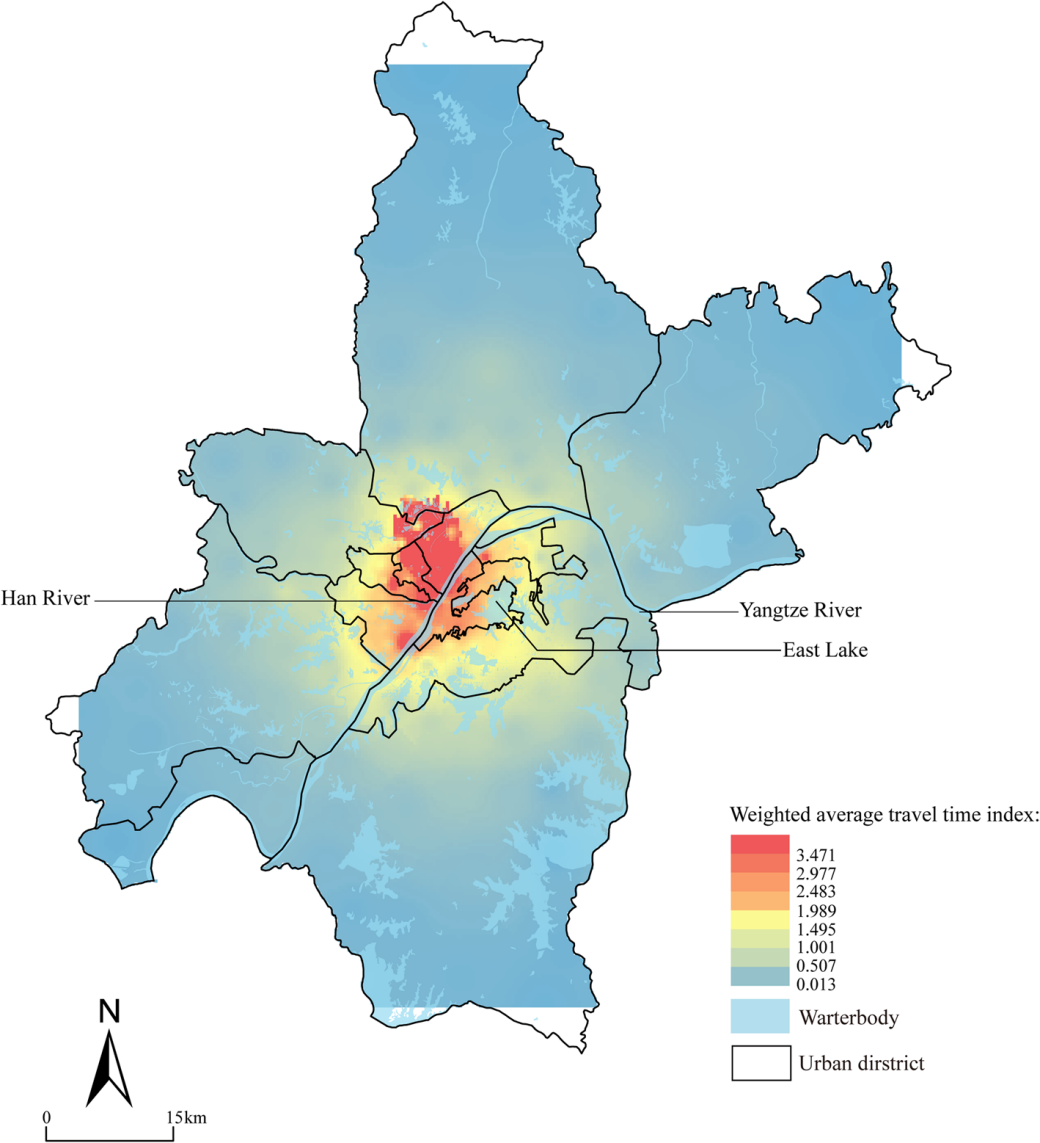
The accessibility of emergency medical facilities

To analyze the accessibility of emergency medical facilities, this study adopts the weighted average travel time method. Since Wuhan did not publish the distribution of epidemic communities, this study takes community service stations as the central point, and the number of beds in different emergency medical facilities as the attraction point. The results show that the accessibility of facilities presents a gradual decay from the central urban area to the distant urban area in multiple circles (Fig. 7). (i) The north of the confluence of Yangtze River and Han River is most accessible in the central city, with a weighted average travel time of 2.289 ~ 3.471; (ii) The east of Yangtze River is the less accessible area in the central city, mainly due to the restriction of the East Lake scenic area; (iii) The accessibility of the city’s suburban area is poorer compared to the central city, with a weighted travel time index of 0.013 ~ 0.579. However, the community density map shows that communities in the less accessible areas are also fewer.

### The degree of facility coverage evaluation

#### Facility number coverage evaluation

The number of emergency medical facilities in each administrative region can reflect the coverage of facilities on a macroscopic level. Combining with the results of the previous spatial analysis of facility distribution, we further used Pearson correlation coefficients to explain the correlation between emergency medical facilities and other factors within the administrative region (Table 2). The results show (Table 3) that the number of the designated hospitals was significantly and positively correlated with the number of general hospitals above level II (*P* ≤ 0.01), the resident population (*P* ≤ 0.05), and the number of confirmed cases (*P* ≤ 0.05). Though the number of the Fangcang shelter hospitals was 1 ~ 2 in each administrative region, Pearson's correlation coefficient showed a significant positive correlation with the population density (*P* ≤ 0.05). This is mainly because the Fangcang shelter hospitals are mostly converted from convention centers and gymnasiums, and the number and size of such places are correlated with the population density of each municipal region.

**Table 2:** Basic information and quantity distribution of emergency medical facilities in Wuhan

Regional position	Region name	Area/km^**2**^	Permanent population (million people)	Population density (million people /km^**2**^)	Secondary and above hospitals’ number	Confirmed cases number	The designated hospitals number	The Fangcang shelter hospitals number
Central area	Jiang An	80.28	96.13	1.1974	14	6563	8	2
	Jiang Han	28.29	72.95	2.5786	8	5242	3	2
	Qiao Kou	40.06	86.71	2.1645	5	6854	2	2
	Han Yang	111.54	64.85	0.5814	4	4691	4	1
	Qing Shan	57.12	52.68	0.9223	7	2804	2	1
	Wu Chang	64.58	127.4	1.9727	14	7551	7	1
	Hong Shan	573.28	160.99	0.2808	9	4718	7	1
Suburban area	Huang Pi	2256.7	96.71	0.0429	1	2117	1	1
	Dong Xihu	495.34	54.11	0.1092	3	2482	1	1
	Cai Dian	1093.17	71.99	0.0659	4	1424	5	2
	Han Nan	287.05	13.16	0.0458	1	1088	1	0
	Jiang Xia	2018.31	89.46	0.0443	2	860	3	1
	Xin Zhou	1463.43	89.48	0.0611	3	1071	3	0

**Table 3:** Pearson correlation coefficient analysis

		Area/km^**2**^	Permanent population (million people)	Population density (million people /km^**2**^)	Secondary and above hospitals’ number	Confirmed cases number
The designated hospitals number	Pearson correlation coefficient	−0.27	.675*	0.22	.810**	.559*
	significance	0.38	0.02	0.46	0.01	0.05
	sample size	45	45	45	45	45
The Fangcang shelter hospitals number	Pearson correlation coefficient	−0.28	0.20	.572*	0.42	0.54
	significance	0.36	0.51	0.04	0.15	0.06
	sample size	15	15	15	15	15

*: 0.01< *P*-*value* ≤ 0.05, **: *P-value* ≤ 0.01

#### Facility service coverage evaluation

It is clear that residents can only go to the designated hospital for the medical treatment process, but not to the Fangcang shelter hospitals by themselves. Therefore, we analyzed the service coverage of emergency medical facilities according to different service recipients.

(i) The designated hospitals’ service coverage

**Fig. 8:**
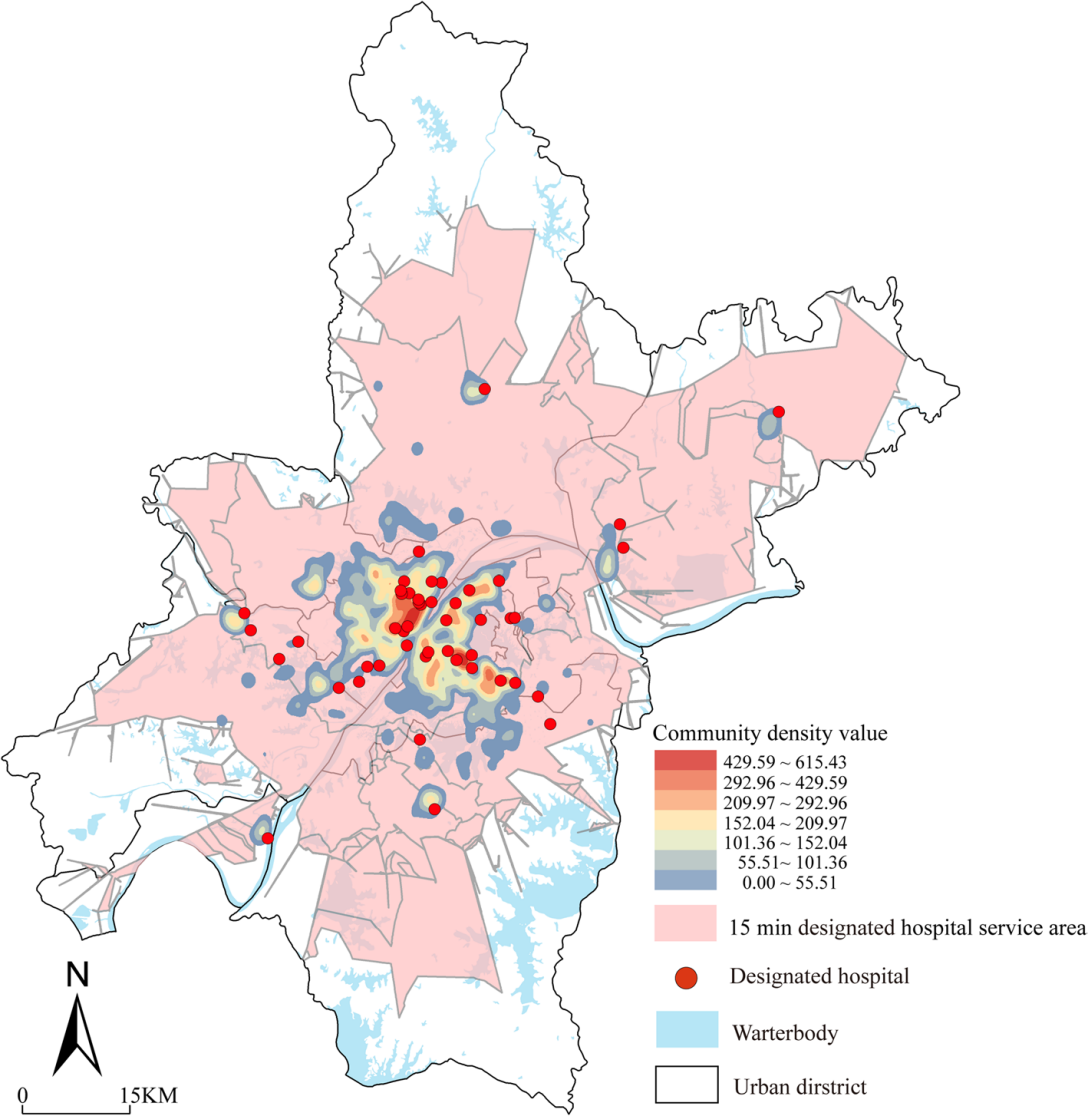
45-min service area of the designated hospital

Many studies have been conducted on the service coverage of general hospitals, indicating that the service range of level II and above hospitals is 40–60 min, and since all the designated hospitals are above level II and 60% of them are level III, and the service range of the designated hospitals is set to 45 min (calculated according to the vehicle speed of 40 km / h) (Liang et al., 2018; Zhao et al., 2020). The results of the 45-min network analysis in ArcGIS show (Fig. 8) that the service area of the designated hospitals cover at least 80% of the administrative area of Wuhan, and the superposed graph of the community density shows that all areas with medium and high values of community density achieve full coverage.

(ii) The Fangcang shelter hospitals’ service coverage

**Fig. 9:**
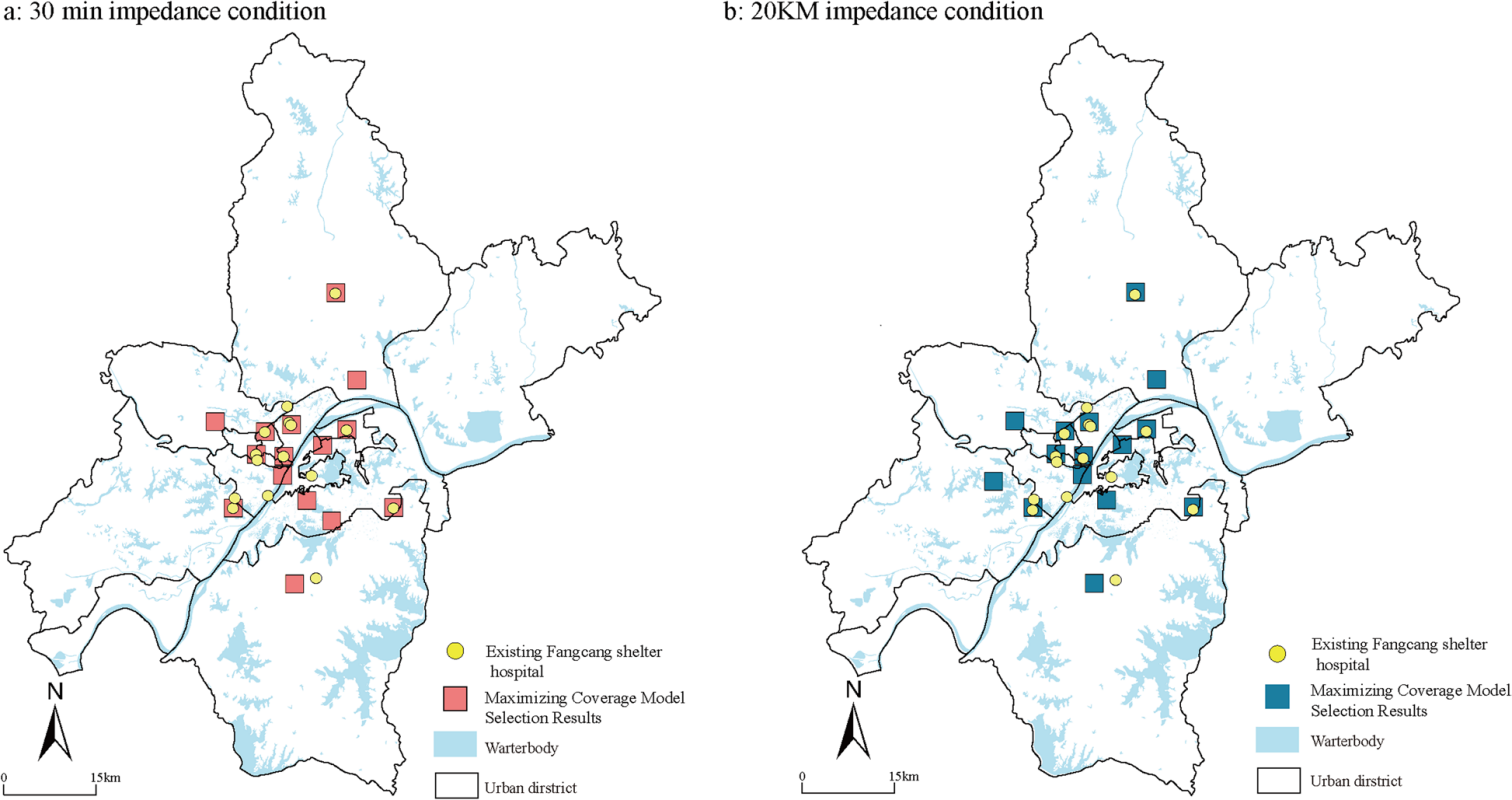
Comparison between the optimization results of the maximum coverage model and the actual distribution of mobile cabin hospitals

Since there are few studies on the service range of the Fangcang shelter hospitals, this study uses a maximized coverage optimization model to analyze the service range of the Fangcang shelter hospitals and evaluate the coverage of community service stations. Firstly, based on the information of the area, grade, and location of the existing Fangcang shelter hospitals, a total of 45 facilities are selected as alternative facility sites. Secondly, by setting impedance conditions, 15 facility sites are selected from them. Then the results are compared with the layout of the existing Fangcang shelter hospitals. As per the average distance between the existing hospitals and community service stations in Wuhan, the distance impedance conditions are set as 10 km, 15 km, and 20 km, and the time impedance conditions are 10 min, 20 min, and 30 min (Table 4). The results show that when the 30 min time impedance is set (Fig. 9), 10 facility sites in the site selection results overlap with the existing Fangcang shelter hospitals’ location and cover 171 community service stations, accounting for 86.35% of the total number. Therefore, the Fangcang shelter hospitals can ensure the emergency demands of transporting confirmed cases from community service stations to the Fangcang shelter hospitals within 30 min.

**Table 4:** Optimization results of maximum coverage model under different impedance conditions

Model name	impedance condition	Number of coincidences locations	Number of coverage demand points	proportion
Maximum coverage model	10 km	6	123	62.12%
	15 km	8	139	70.20%
	20 km	8	150	75.76%
	10 min	6	123	62.12%
	20 min	8	152	76.77%
	30 min	10	171	86.36%

## Discussion

The background of globalization has provided great convenience for the spread of epidemics and pose a severe test for urban safety. Emergency medical facilities, as one of the most effective measures for public health emergencies, are necessary to be prepared (Luo et al., 2020). With the help of Emergency medical facilities and the implementation of strict epidemic prevention policies and measures, the epidemic in Wuhan has been effectively controlled. Therefore, we analyze the layout characteristics and rationality of Wuhan’s emergency medical facilities from a spatial perspective during the epidemic period using data published by the Wuhan Health Commission and Baidu maps. We found that previous studies only focused on the role of the Fangcang shelter hospitals in the epidemic, (Dai et al., 2020; Yan et al., 2020; Lai et al., 2020) but ignored the integrity and linkages of emergency medical facilities. Therefore, a more comprehensive perspective on the role of the layout of emergency medical facilities in controlling epidemic deserves further exploration.

In the overall distribution, all facilities form the “one center, two circles” pattern, and the results of the accessibility and coverage of facilities also prove the effectiveness of this pattern. The “one center” makes full use of the existing medical resources in the city, and the central city’s location facilities can rapidly mobilize other medical resources, forming an effective first line of defense against public health emergencies. The “two circles” model establishes the Fangcang shelter hospitals in suburban areas and makeshift hospitals in distant urban areas to reduce the impact of surrounding residents while forming a closed-loop for emergency treatment within the city. Due to the abrupt epidemic, there are still a small number of Fangcang shelter hospitals that have to be located in the center of the city to quickly layout emergency medical facilities. Although these Fangcang shelter hospitals have a certain buffer zone from the surrounding buildings, they may still bring inconvenience to the residents’ psychology and life. Then more consideration should be given to reserving land for the construction of emergency medical facilities in the urban planning process, while the criteria for the layout of emergency medical facilities should be clarified in the planning policies of different levels. For example, the process of integration, adjustment, and participation should be emphasized in the formulation of spatial planning, and the overall requirements of medical and health-related planning should be clarified. Furthermore, the recent planning schemes for different emergency event states should be prepared, the principles and standards for the layout of emergency medical facilities should not only be determined in the urban master plan but also the indicators and uses of the city’s white areas should be ensured to provide better locations for the construction of similar facilities in the future (Huang Jingnan et al., 2014; Xiang, 2003). The medical and health Specialized planning should avoid focusing only on the spatial configuration of medical facilities, but also formulate the emergency functions that different facilities should undertake according to the layout characteristics of different medical facilities, and improve the multi-sectoral collaboration and linkage sharing mechanism.

**Fig. 10:**
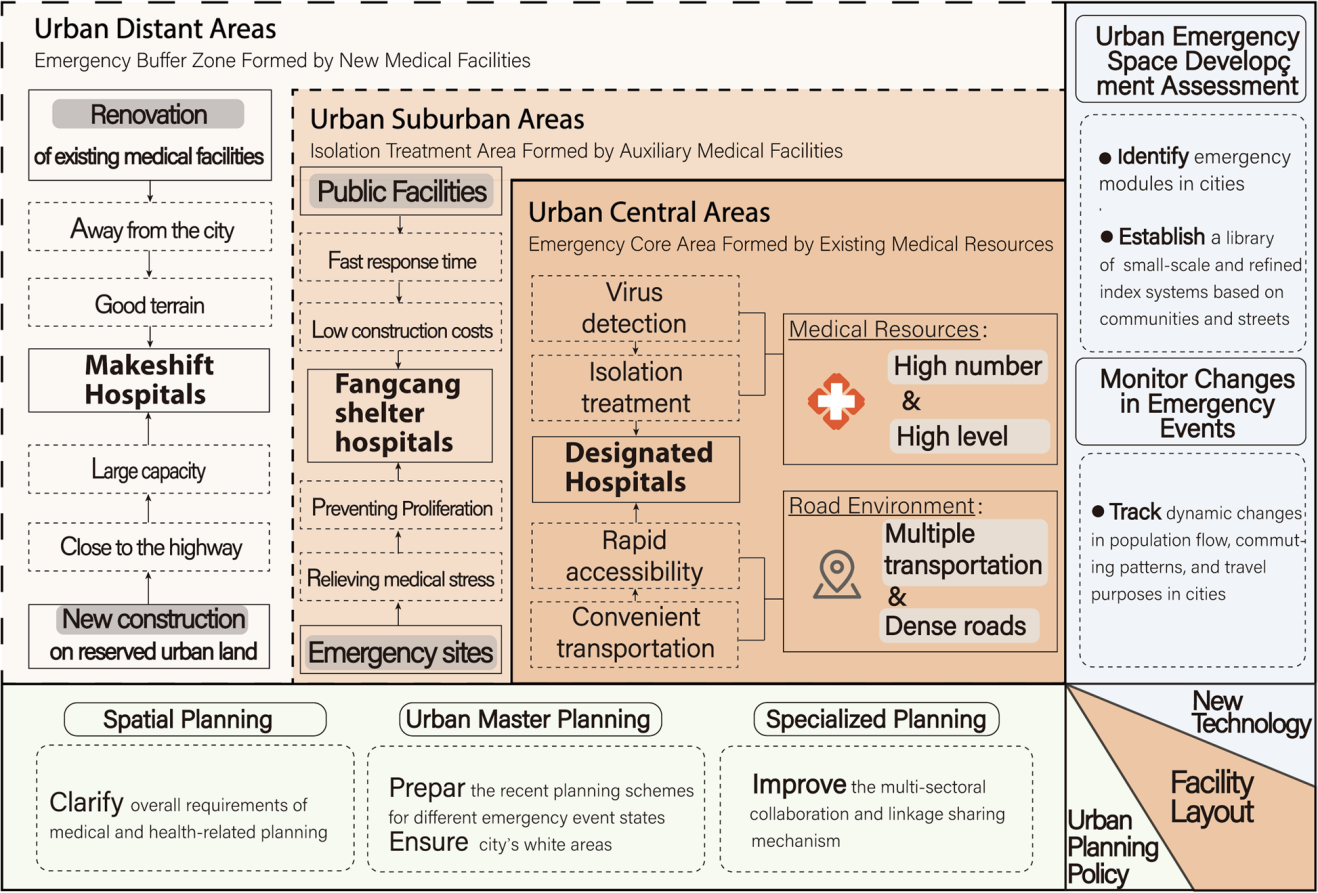
Layout optimization suggestions of urban emergency medical facilities

According to the evaluation results, the effectiveness of the emergency timeliness of the facilities is weak. The study finds that the measure of laying out the makeshift hospitals first in Wuhan did not fully release the timeliness of the emergency medical facilities and that the emergency timeliness was not fully released until the Fangcang shelter hospitals were put into operation. Therefore, artificial intelligence and machine learning technologies are a new way to solve this problem. In terms of urban emergency space development assessment, due to the differences of zoning units of different scales and locations in cities in response to emergencies, a library of small-scale and refined index systems based on communities and streets should be established. Combined with urban emergency planning, the use of video analysis and other technologies can identify emergency modules in cities that can be used as the layout of emergency medical facilities and assess the development of urban emergency space through basic development indicators such as volume ratio, population density, and road network density, so that countermeasures can be proposed according to different levels of public health emergencies. In terms of dynamic changes in the development of emergency events: the dynamic changes in the regional patterns of urban population flow, commuting patterns, and travel purposes can be continuously tracked with the help of big data to identify further changes in the strength of connections and functional characteristics between cities on a macroscopic level, and to propose targeted early warnings and countermeasures for cities to respond to similar public health emergencies in the future. At present, COVID-19 has been brought under control in China, but there are still intermittent rebounds in some cities. In order to control the epidemic, some cities will adopt the urban static management strategy during the period of rapid increase of the epidemic. At this stage, the daily medical problems of residents need to be paid attention to. Therefore, it is necessary to consider different types of medical treatment groups such as pregnant women, the elderly, and patients with chronic diseases. According to their diagnosis and treatment conditions, people-oriented urban planning ideas should be added to the patient care system. In addition, different disciplines such as urban planning, public management, and medical care should establish an interdisciplinary emergency management department to join the decision-making of urban planning and management, so as to promote multi-disciplinary integration and improve the city’s ability to respond to such events (Fig. 10).

## Conclusion

This paper applies mathematical statistics, spatial analysis and other methods to explore its spatial agglomeration, distribution characteristics, and discusses evaluation of the emergency timeliness, spatial accessibility, facility coverage. The researches show that the emergency medical facilities are symmetrically distributed along the “northeast - southwest” axis, formed a pattern of “central cluster + peripheral dispersion” and the spatial agglomeration characteristics. In the middle and late stages of the COVID-19 epidemic， timeliness evaluation of emergency shows efficient with the opening of mobile cabin hospitals. The overall spatial accessibility is relatively convenient, forming a trend of gradual decline from high accessibility in central urban area to low accessibility circle in remote urban area. The overall coverage of the facilities is sufficient. The distribution of various medical facilities takes into account the medical level, resident population, population density and other objective factors in each administrative region; designated hospitals and shelter hospitals can cover more than 80% of the administrative area and the number of community service centers. This study provides a reference for the layout of emergency medical facilities in response to serious public health emergencies, and also provides help for urban planning and urban management to improve the resilience of hospitals.

There are some limitations to our work. First, this study mainly considers the geographic and spatial data related to the layout of emergency medical facilities, and other factors like the influence of the policy measures, the urban development level, and the patients’ access habits should be more considered in further studies. Secondly, since the Wuhan government did not publish the neighborhoods where the confirmed cases were located, this study treats all neighborhoods and community service stations as demand points, making the sample size larger. The findings of this study may provide a reference for cities in other countries to cope with similar challenges. In the future, it will be an important research direction to explore the normalization layout of urban emergency medical facilities under the perspective of emergency disaster relief. In addition, in response to the shortage of medical supplies and medical personnel exposed to this epidemic, it is also worth exploring how to establish an urban emergency logistics distribution system and linkage system on time through big data, smart cities, and other technological means in the future.

## Data Availability

All data generated or analyzed during this study are included in this published article. All the data used for several analyses are freely available and the resources are mentioned within the paper.

## Abbreviations

COVID-19: Corona Virus Disease 2019
PAHO: Pan American Health Organization
PPRR: Prevention and mitigation (P), Preparation and planning (P), Response and relief (R), Recovery (R)
NAT: Nucleic acid testing
H2SFCA: Hierarchical two-step floating catchment area
